# Supplementary material for: Targetable lesions and proteomes predict therapy sensitivity through disease evolution in pediatric acute lymphoblastic leukemia
Source: Nat Commun. 2023 Nov 21;14:7161. doi: 10.1038/s41467-023-42701-9 (PMC10663560; doi:10.1038/s41467-023-42701-9)
Supplement: Supplementary file 1 — Supplementary Information [file 41467_2023_42701_MOESM1_ESM.pdf]

Targetable lesions and proteomes predict therapy sensitivity through disease evolution in pediatric acute lymphoblastic leukemia

Amanda C. Lorentzian[1,2,] Jenna Rever[1,2], Enes K. Ergin[2,3], Meiyun Guo[1,2], Neha M. Akella[1,2], Nina Rolf[1,2], C. James Lim[1,2], Gregor S.D. Reid[1,2], Christopher A. Maxwell[1,2,4], Philipp F. Lange[2,3,4]

Affiliations:
1. Department of Pediatrics, University of British Columbia, Vancouver, CANADA
2. Michael Cuccione Childhood Cancer Research Program at the BC Children's Hospital Research Institute, Vancouver, CANADA
3. Department of Pathology, University of British Columbia, Vancouver, CANADA

Supplementary Information

Supplementary Figure S1 BCCH cohort overview

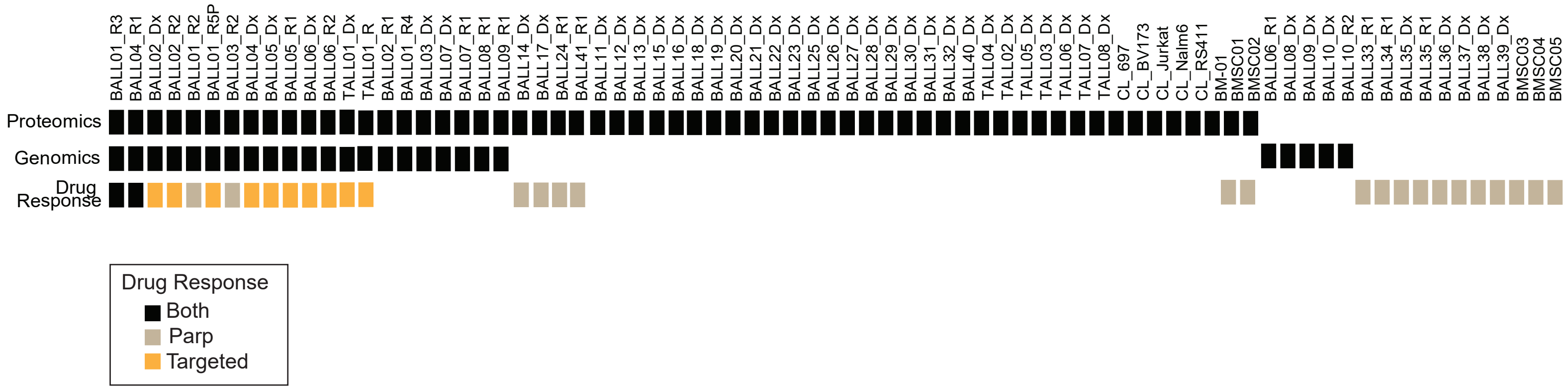

Supplementary Figure S1 BCCH cohort overview
Overview of all the samples described in the manuscript annotated by which analysis each specimen was included in (black). Specimens that were included only in the targeted inhibitor analysis are annotated in orange, and those that were only included in the PARP inhibitor analys are anotated with light brown.

Supplementary Figure S2: Genomic stability in paired ALL specimens from the BCCH cohort

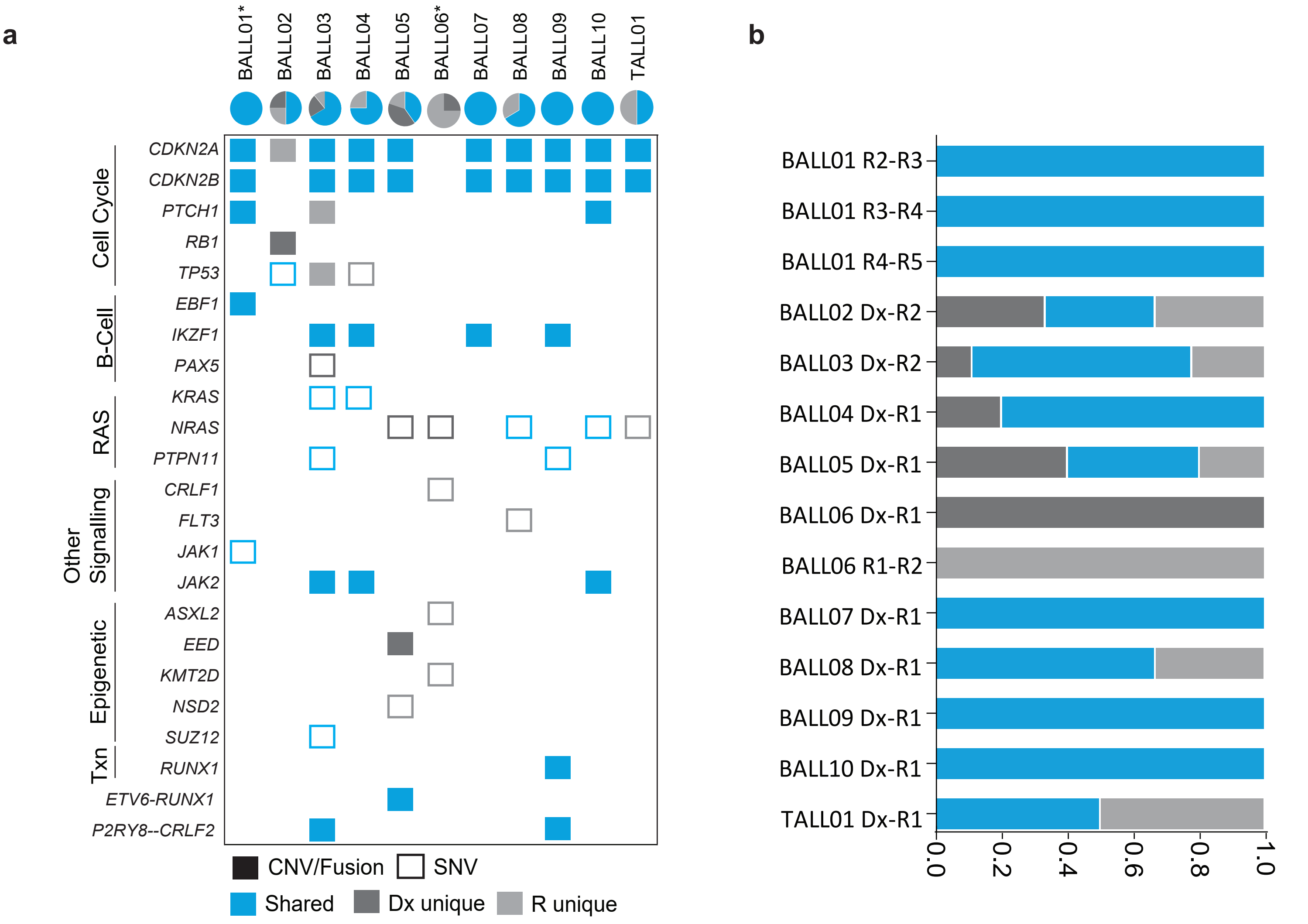

Supplementary Figure S2: Genomic stability in paired ALL specimens from the BCCH cohort. a Mutated gene products identified through targeted DNA/RNA-fusion sequencing of paired diagnosis (Dx) and relapse (R) samples. Mutations detected in both Dx and R samples are represented by blue boxes, mutations unique to Dx are dark grey, and R unique are light grey. CNVs are full boxes and SNVs are outlined boxes. The pie diagrams at the top summarize the number of mutations for each category for each patient. An asterisk indicates multi timepoint patients; BALL01 R2-R3-R4-R5-R5P, BALL02 Dx-R1-R2. **b** Bar graphs for each patient at each timepoint illustrate the fraction of variants that are unique to diagnosis (Dx Unique, dark grey bars), unique to relapse (R unique, light grey bars), or shared (blue bars).

Supplementary Figure S3: Genomic lesions detected in paired ALL specimens from the BCCH cohort

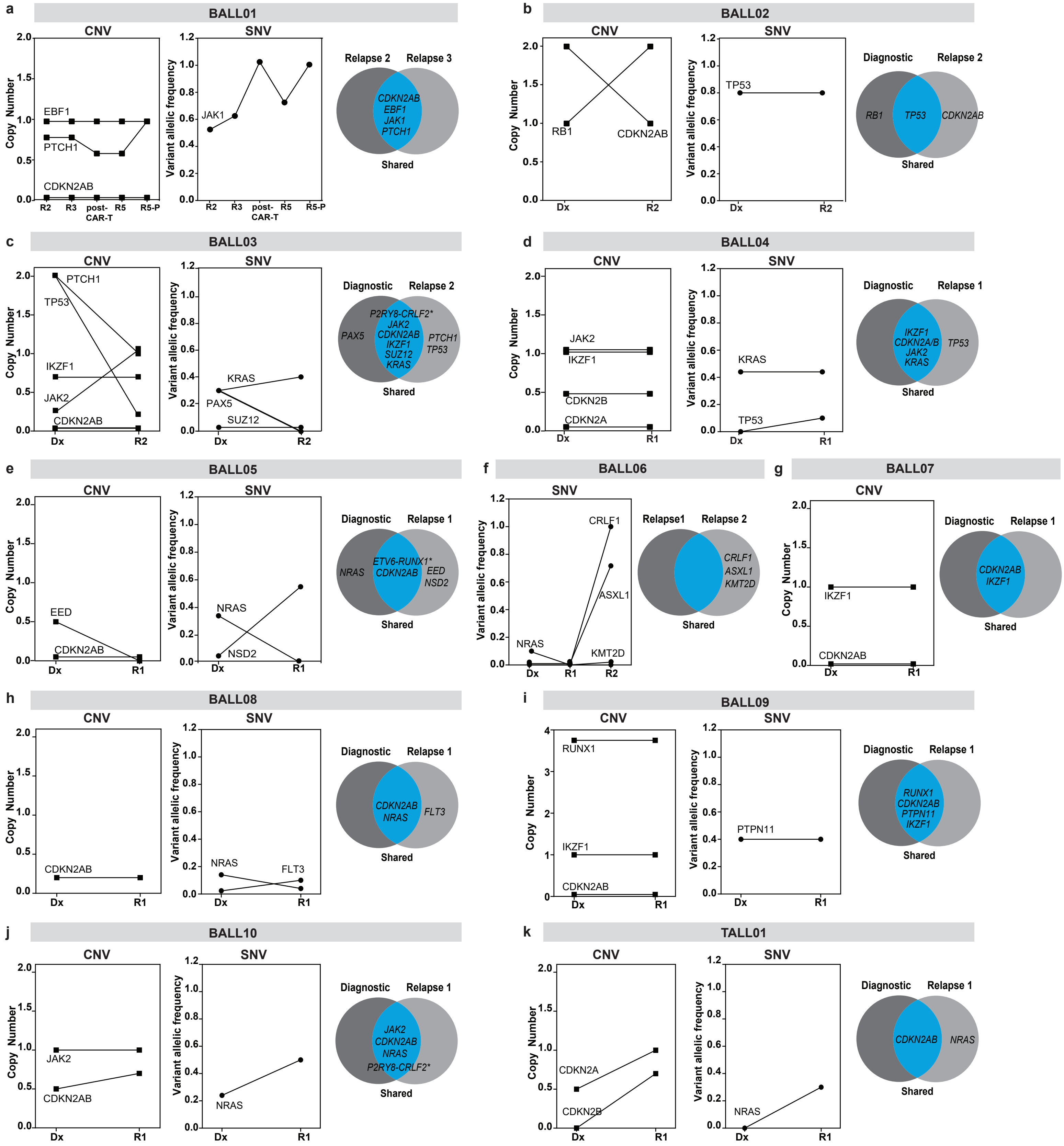

Supplementary Figure S3: Genomic lesions detected in paired ALL specimens from the BCCH cohort
a-k. Line graphs for each patient treated at BCCH represent genomic lesions identified through targeted NGS in samples collected through disease progression. Only abnormal variants are plotted. CNVs are plotted by the number of copies detected and SNVs are plotted by the allelic frequency. Venn diagrams for each patient display variants that are unique to diagnosis (Dx unique), unique to relapse (R unique), or shared between samples in a progression series.

Supplementary Figure S4: Genomic lesions detected in paired ALL specimens from the SJH cohort

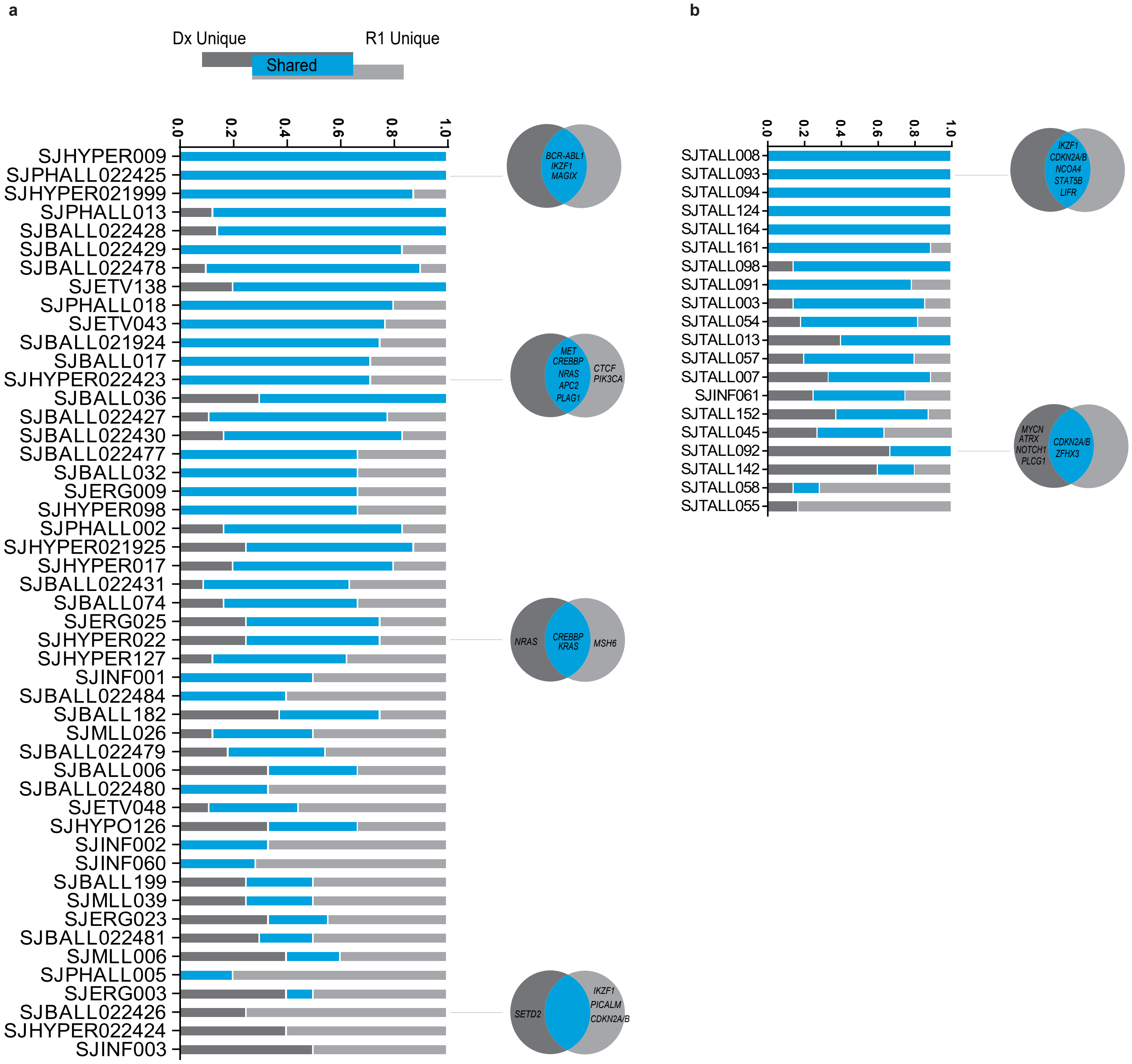

Supplementary Figure S4: Genomic lesions detected in paired ALL specimens from the SJH cohort
a Bar graphs for each patient with B-ALL treated at SJH (n=49) illustrate the fraction of variants that are unique to diagnosis (Dx Unique, dark grey bars), unique to relapse (R unique, light grey bars), or shared (blue bars), identified through whole genome sequencing in samples collected through disease progression. Venn diagrams are shown for selected patients to display the distribution of variants in a progression series. **b** Bar graphs for each patient with T-ALL treated at SJH (n=20) illustrate the fraction of variants that are unique to diagnosis (Dx Unique, dark grey bars), unique to relapse (R unique, light grey bars), or shared (blue bars), identified through whole genome sequencing in samples collected through disease progression. Venn diagrams are shown for selected patients to display the distribution of variants in a progression series.

Supplementary Figure S5: Dynamics of genomic lesions detected in the SJH and BCCH cohorts

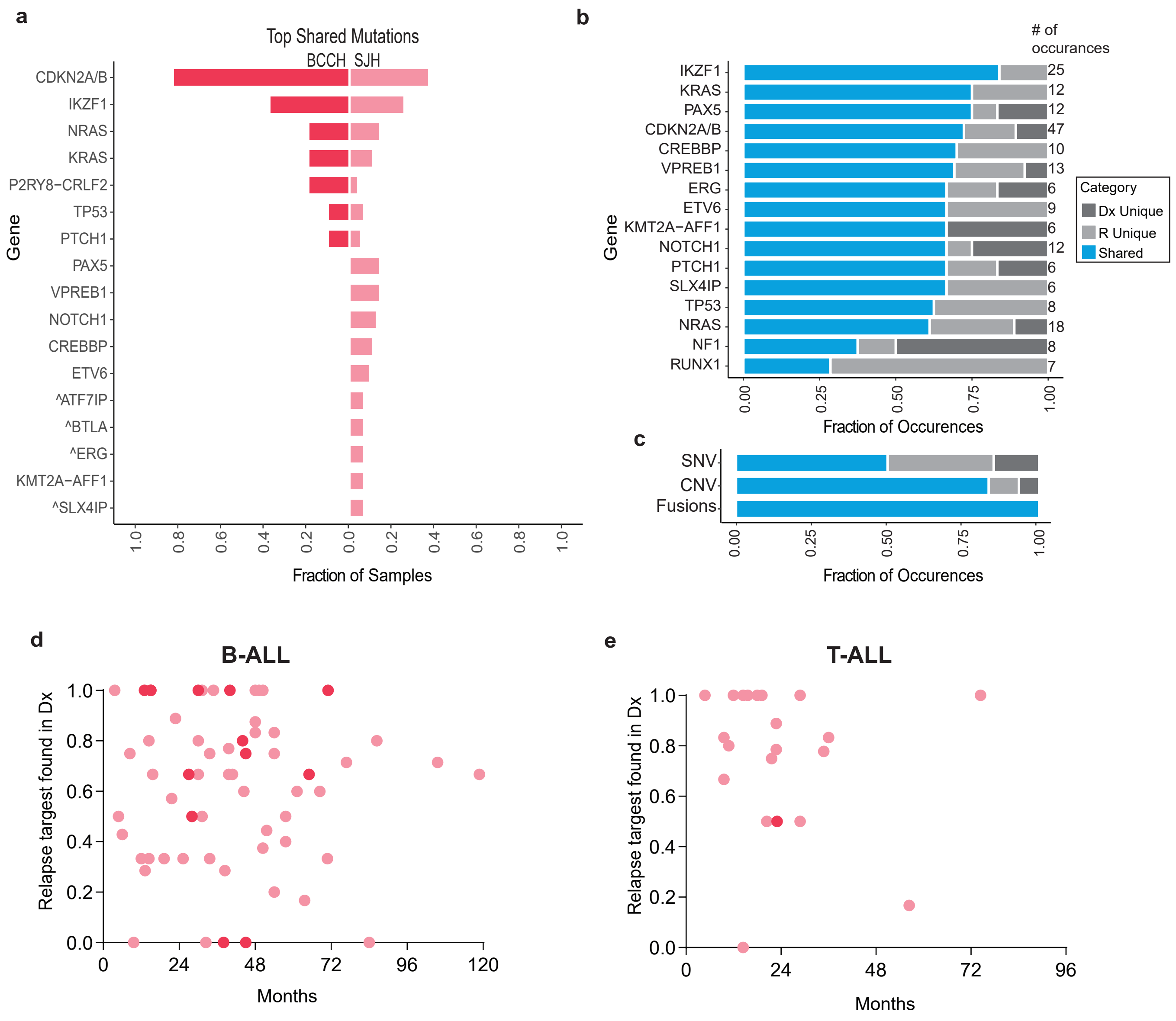

Supplementary Figure S5: Dynamics of genomic lesions detected in the SJH and BCCH cohorts. a Bar plot for the most frequent variants (top 17) detected in both Dx and paired R samples (shared) plotted as the fraction of samples containing the shared variant in each cohort (n=11 patients from BCCH (light pink), n=69 patients from SJH(red)). Variants were detected in the BCCH cohort through targeted NGS while variants were detected in the SJH cohort through whole genome sequencing. Thus, variants that cannot be detected in the BCCH samples with the targeting sequencing assay are indicated with ^. **b** Bar plot for the most prevalent genes (top 16) detected in the combined BCCH and SJH cohorts. Variants are categorized as shared (blue), Dx unique (dark grey) or R unique (light grey) and plotted as the fraction of occurrences. The total number of times the gene was identified is displayed on the right end of the bar. **c** Bar plot classifying variants (SNV, CNV, Fusion) as shared (blue), Dx unique (dark grey) or R unique (light grey) and plotted as the fraction of occurrence in the BCCH cohort. **d** Dot plot for fraction of shared variants versus time to relapse for 59 B-ALL patients (n= 10 patients from BCCH represented by light pink circle, n= 49 patients from SJH represented by red circles). **e** Dot plot for fraction of shared variants versus time to relapse for 21 T-ALL patients (n= 1 patient from BCCH represented by light pink circle, n= 20 patients from SJH represented by red circles).

Supplementary Figure S6- BCCH Proteome Cohort- Batch Correction and Data Quality

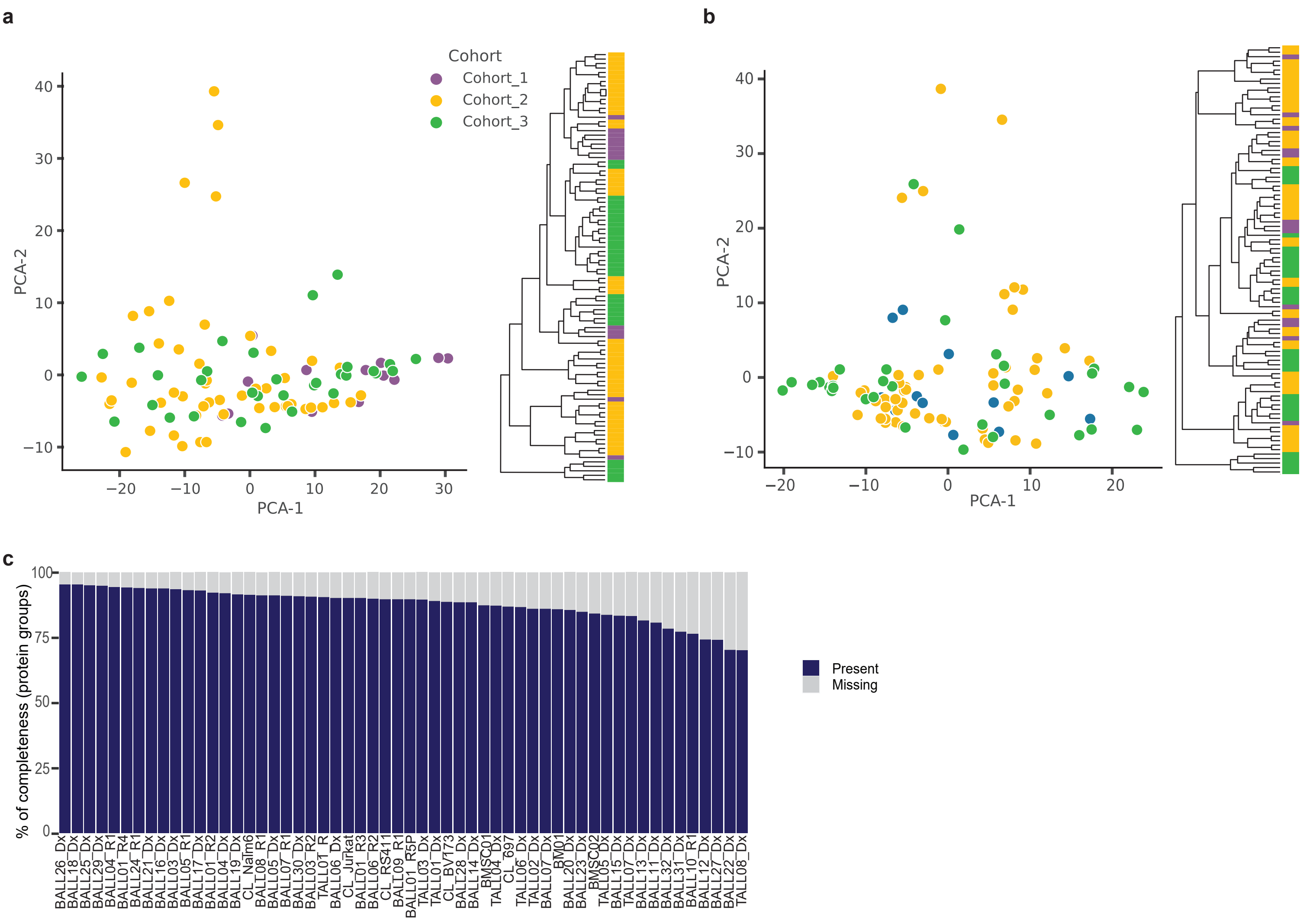

Supplementary Figure S6: BCCH Proteome Cohort- Batch Correction and Data Quality. a (Left) PCA of proteins (n=2995 proteins) identified in all samples (n=56 samples) prior to batch correction. The points are colored by cohort; cohort 1 in purple, cohort 2 in yellow, and cohort 3 in green. (Right) Hierarchical clustering of the 2995 proteins prior to batch correction scaled by min/max. The dendrogram indicates the clustering of the samples and the color bar indicates the cohort the sample was from. **b** (Left) PCA of proteins (n=2995 proteins) identified in all samples (n=56 samples) after batch correction. The points are colored by cohort; cohort 1 in purple, cohort 2 in yellow, and cohort 3 in green. (Right) Hierarchical clustering of the 2995 proteins after batch correction scaled by min/max. The dendrogram indicates the clustering of the samples and the color bar indicates the cohort the sample was from. **c** Visual of data completeness in all of the samples after batch correction. Percentage of completeness is represented in blue and missingness is represented in grey.

Supplementary Figure S7: PCA clusters samples based on sample type

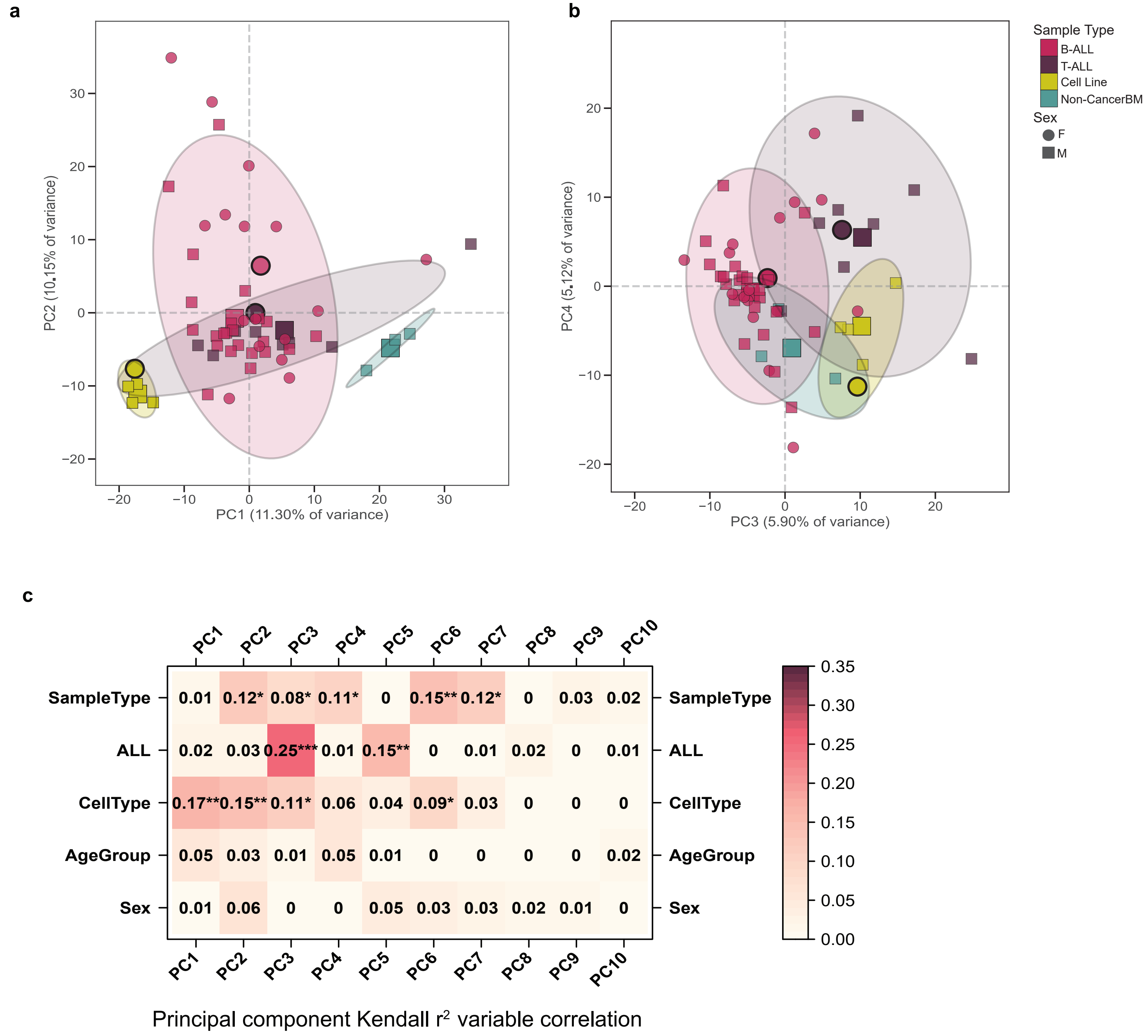

Supplementary Figure S7: PCA clusters samples based on sample type. a component analysis (PCA) of the BCCH proteome cohort (n=56 samples) based on PC1 vs PC2 of quantified proteins. The color represents sample type and shapes represent sex. **b** Principal component analysis (PCA) of the BCCH proteome cohort (n=56 samples) based on PC3 vs PC4 of quantified proteins. The color represents sample type and shapes represent sex. **c** Kendall correlation to identify the contribution of each metavariable per PC (0.0001****, 0.001***, 0.01**,0.05*) to indicate the significance of the correlation. The values are colored from low correlation value (yellow) to high correlation value (purple).

Supplementary Figure S8: Description of proteomic data filtering pipeline and quality assessment

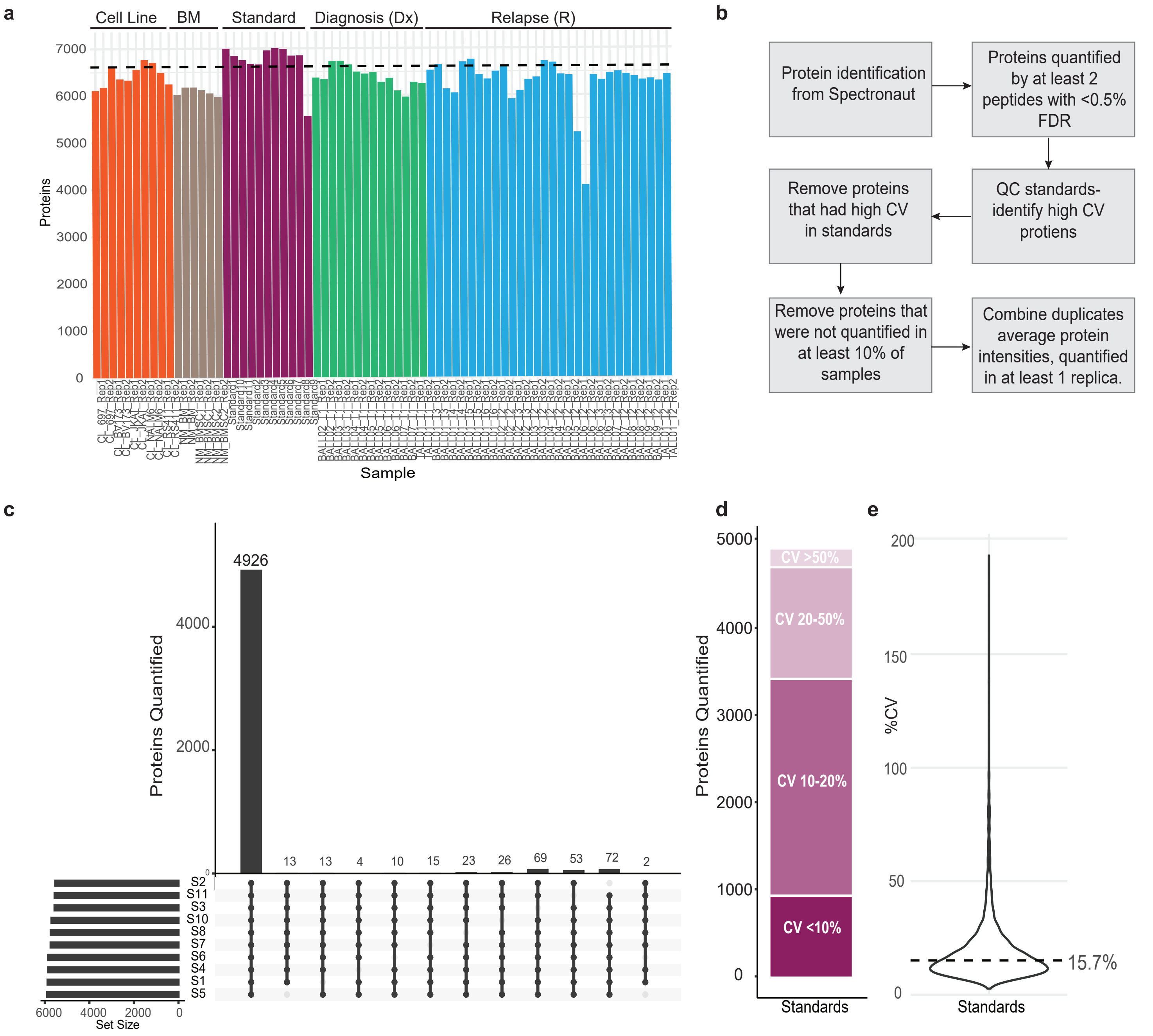

Supplementary Figure S8: Description of proteomic data filtering pipeline and quality assessment. a Total protein groups identified in each sample (n=71 samples), prior to any filtering. The category of the sample type is listed across the top of each group. **b** Diagram of the filtering workflow to attain the final proteomics dataset utilized for subsequent analyses. **c** Upset plot for ten of the eleven standards (standard nine was removed due to a clear technical issue). Numbers reported are based on protein groups quantified by a minimum of two peptides at a precursor q-value threshold of 0.5% FDR. **d** The cv for protein quantity across the ten standards was assessed and plotted by percent coefficient of variation (CV). The darkest purple bar at the bottom represents the number of proteins with a CV of less than 10% and so on, with the lightest bar at the top representing the number of proteins with a cv greater than 50%. This fraction of proteins with cv >50% was removed from the remaining data with the assumption these are unstably quantified between samples. **e** A violon plot demonstrating the median CV of protein quantification across the ten standards (including those >50% CV). Dashed line indicates the median CV (15.7%).

Supplementary Figure S9: Evaluation of individual sample data quality

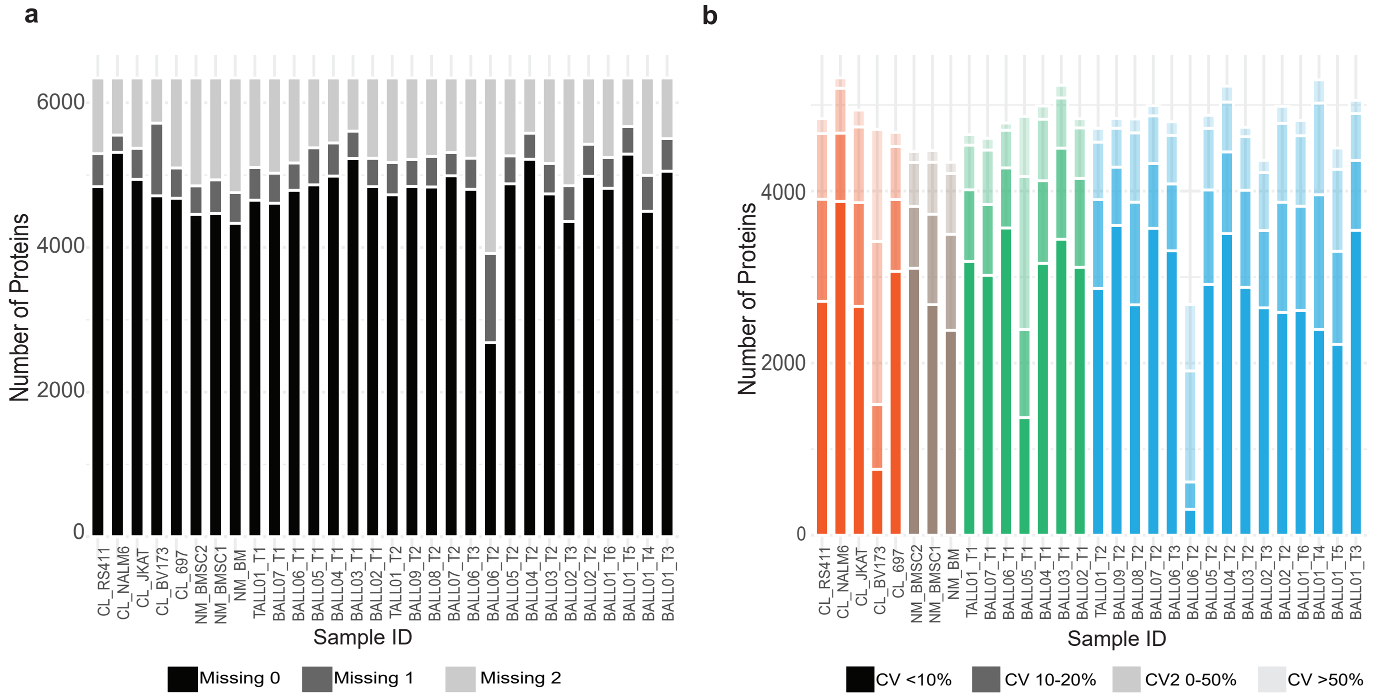

Supplementary Figure S9: Evaluation of individual sample data quality. a Bar plot to demonstrate data completeness between replica of each sample (n=30 samples). Proteins that were identified in both replica are represented in black, dark grey represents proteins that were only identified in one of the replica, and light grey represents proteins that were entirely missing from the pair. **b** CV between replica for the 30 samples is represented as described in panel S5 D; The darkest color bar at the bottom represents the number of proteins with a CV of less than 10% and so on, with the lightest bar at the top representing the number of proteins with a cv greater than 50%.

Supplementary Figure S10: Statistical analysis of paired samples

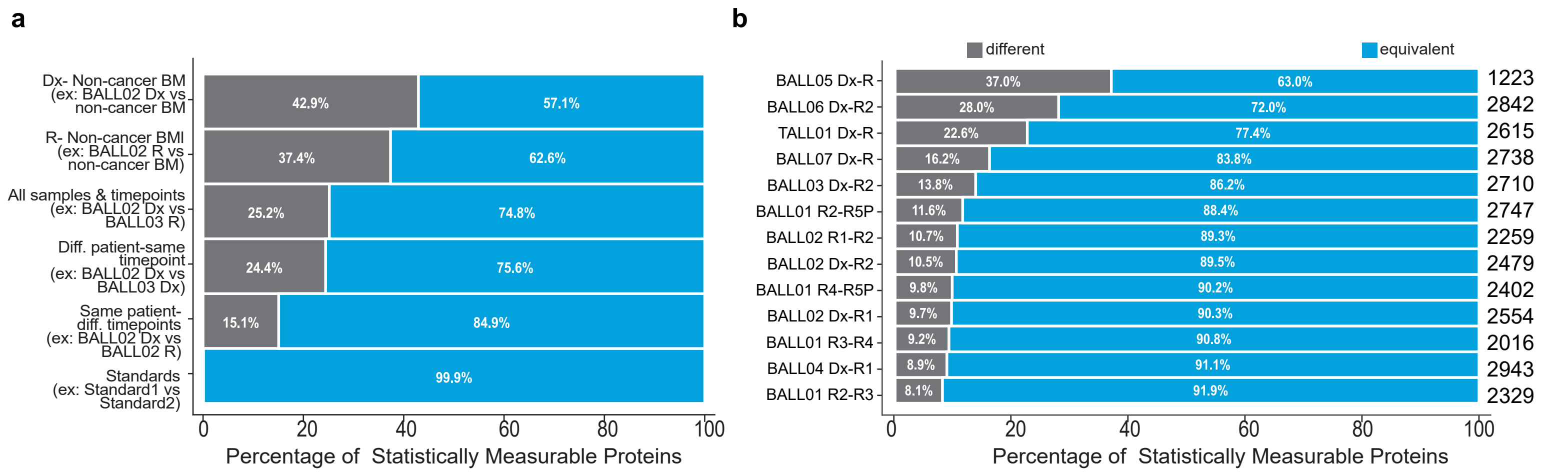

Supplementary Figure S10: Statistical analysis of paired samples. a Summary of tests for differential expression and equivalence between different groups and pairings. Proteins that are statistically equivalent are represented in blue (Two-one-sided t-test (TOST) for equivalence, boundaries between log2FC<-1 and log2FC>1), proteins that are statistically different are represented in grey (student's t-test adjusted FDR<0.05, log2FC>1). The bar represents the mean equivalence or difference of all protein expression for each pairing within the group. The number of comparisons in each group from top to bottom are n= 21 pairs, n= 45 pairs, n= 76 pairs, n= 98 pairs, n= 17 pairs, and n= 210 pairs. **b** Similar representation for each individual patient pairing of the group "Same patient-diff timepoints" group. The numbers on the right of each bar indicate how many statistically measurable proteins were in each pairing.

Supplementary Figure S11 Statiscally different proteins between Dx-R

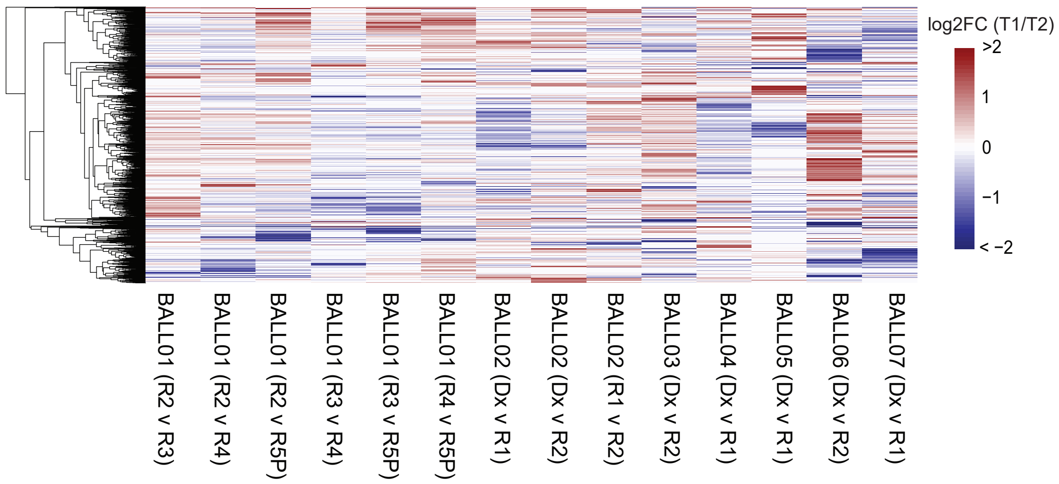

Supplementary Figure S11 Statistically different proteins between Dx-R
The results for statistically different proteins for each pair. The list of significantly different proteins (n= 2637 proteins) for Dx vs R was derived from the proteins that were deemed significantly different from each Dx-R pair (student's t-test p-value adjusted FDR<0.05, log2FC >1, log2FC <-1). Since BALL01 does not have a Dx, BALL01 was not included as a comparison to create the list of significantly different proteins. of this list. The heatmap represents the log2FC of timepoint 1 (T1) / timepoint 2 (T2) and is clustered row-wise by the ward.D method for hierarchical clustering.

Supplementary Figure S12: FACS isolation of mature B-cells

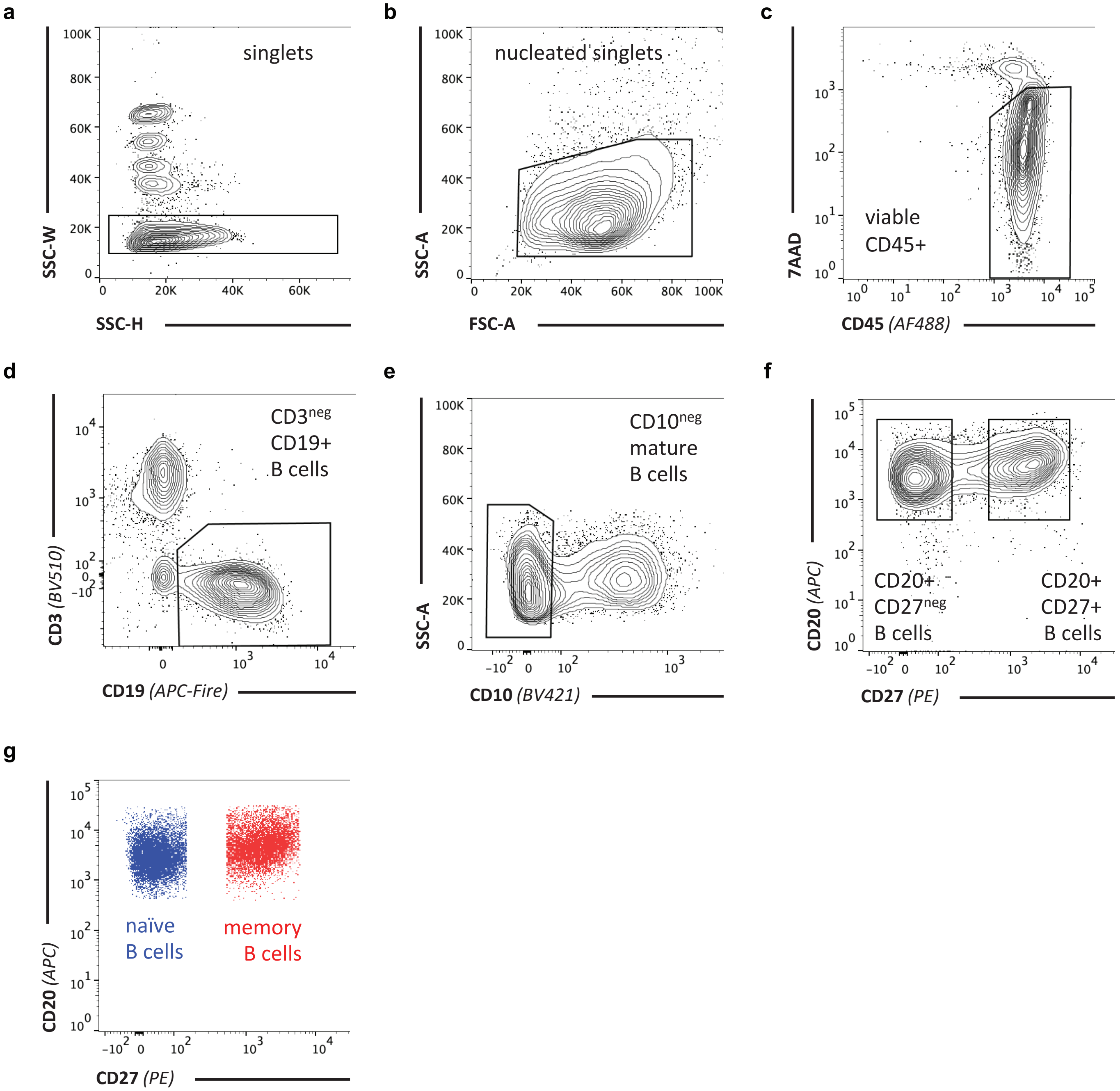

Supplementary Figure S12: FACS isolation of mature B-cells
Gating strategy to isolate naïve and memory B-cells. **a-c** Viable singlet cells are selected. **d** CD19+ B-cells are separated from the CD3+ T-Cells. **e** CD10- mature B-cells are separated from CD10+ immature B-cells. **f-g** Naïve B-cells are separated from memory B-cells are separated based on CD27 expression.

Supplementary Figure S13: Significant cancer associated proteins (CAPs)

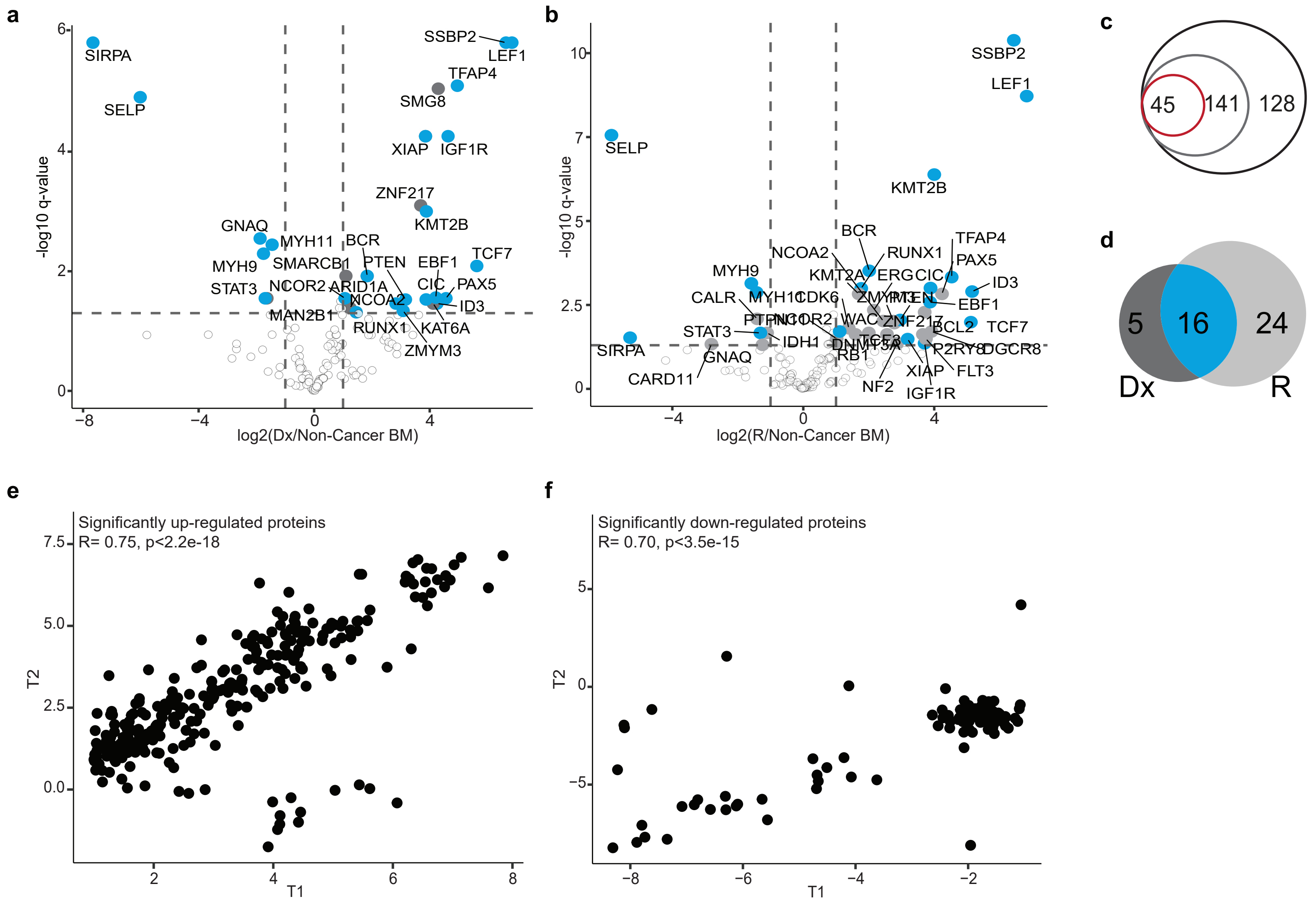

Supplementary Figure S13: Significant cancer associated proteins (CAPs). a 141 proteins were tested using LIMMA (log2FC>1, p-value adjusted FDR<0.05); Initial Diagnosis (Dx) samples vs. Non-cancer BM samples and **b** Relapse (R) vs Non-cancer BM samples. Proteins that are significantly over expressed in both Dx and R are colored in blue and those that are unique to either Dx or R are colored in grey. **c** From the list of 269 pediatric cancer driving proteins (black circle), 141 proteins were detected in our data (gray circle) and 45 proteins were deemed significant (red circle). **d** Sixteen of the over-abundant proteins were shared between Dx and R (bottom). **e** Of the 45 proteins that were over-abundant at Dx, the protein abundancefor each protein at each timepoint was plotted as timepoint 1 (T1) vs timepoint 2 (T2) (calculated as the log2(protein abundance/the average protein abundance in the non-cancer bone marrow (BM) samples). A Pearson's r correlation was calculated for the entire dataset. **f** Of the 45 proteins that were under-abundant at Dx, the protein abundance for each protein at each timepoint was plotted as timepoint 1 (T1) vs timepoint 2 (T2) (calculated as the log2(protein abundance/the average protein abundace in the non-cancer bone marrow (BM samples). A Pearson's r correlation was calculated for the entire dataset.

Supplementary Figure S14: RNA stability in paired Dx-R ALL patients from BCCH and TARGET cohorts

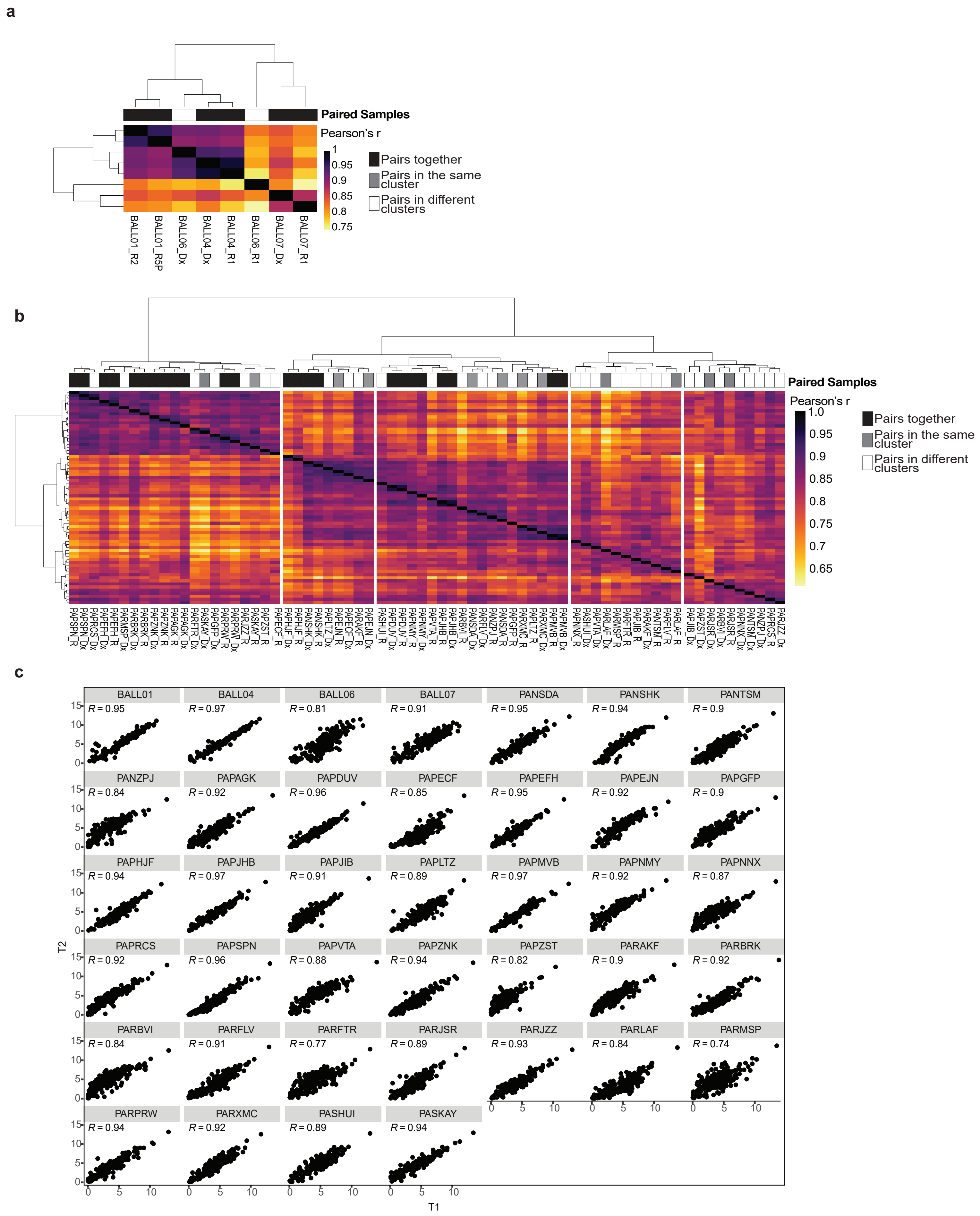

Supplementary Figure S14: RNA stability in paired Dx-R ALL patients from BCCH and TARGET cohorts. a Pearson correlation of 4 paired patients (average of n=2 replica) from the BCCH Cohort. Sample pairs that clustered directly next to each other are annotated in black, sample pairs that were in the same major cluster but not directly next to each other are annotated with grey, and those that were in different major clusters are annotated with white. **b** Pearson correlation of 35 paired (70 samples total) pediatric ALL patients based on publicly available whole transcriptome data from the TARGET initiative. Sample pairs that clustered directly next to each other are annotated in black, sample pairs that were in the same major cluster but not directly next to each other are annotated with grey, and those that were in different major clusters are annotated with white. **c** Pearson correlation matrix for the combined TARGET and BCCH patient pairs plotted as timepoint 1 (T1) vs timepoint 2 (T2) for the cancer-associated proteins (CAPs).

Supplementary Figure S15: Summary of PARP1 and γH2Ax immunofluorescence data

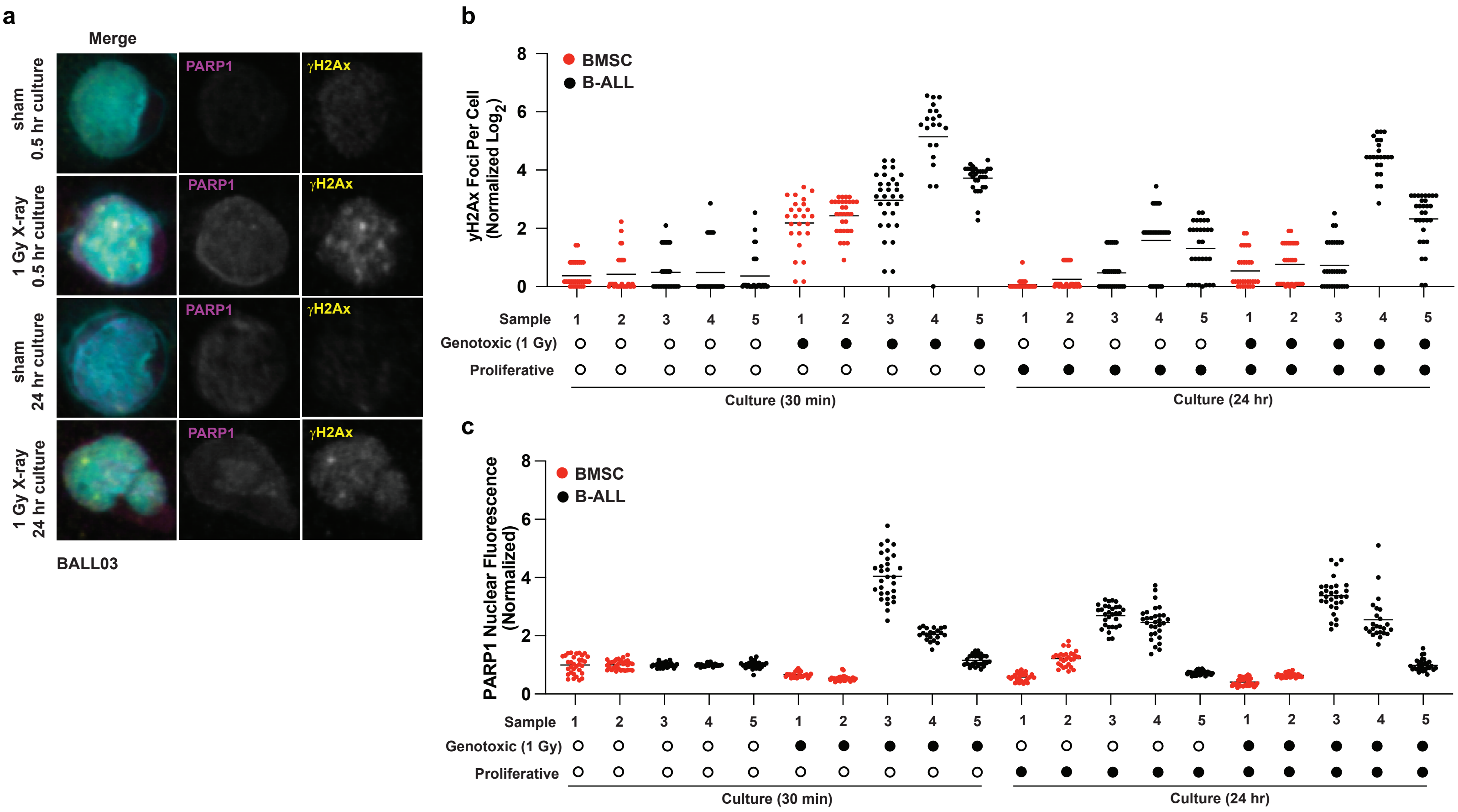

Supplementary Figure S15 Summary of PARP1 and γH2Ax immunofluorescence data. a Representative image showing immunoflourescence staining of γH2Ax and PARP1 individually, and merged with Hoechst nuclear stain for primary sample BALL03-R2. Samples were treated with 1 Gy X-irradiation or sham conditions, and co-cultured with hTERT-MSCs for 30 minutes or 24 hours after treatment. **b** Log2 γH2Ax foci per cell normalized to sham treatment at 30 minutes, quantified from immunfluorescence analysis of 2 BMSC (red) samples and 3 B-ALL (black). Individual primary samples are indicated by number (1=BMSC02, 2=BMSC05 3= BALL04-R1, 4=BALL01-R2, 5=BALL03-R2) (n=30 cells per sample from one individual experiment). Samples were treated with 1 Gy X-irradiation or sham conditions, and co-cultured with hTERT-MSCs for 30 minutes or 24 hours after treatment. **c** Average PARP1 nuclear fluorescence per cell normalized to sham treatment at 30 minutes, quantified from immunfluorescence analysis of 2 BMSC (red) samples and 3 B-ALL (black). Individual primary samples are indicated by number (1=BMSC02, 2=BMSC05 3= BALL04-R1, 4=BALL01-R2, 5=BALL03-R2) (n=30 cells per sample from one individual experiment). Samples were treated with 1 Gy X-irradiation or sham conditions, and co-cultured with hTERT-MSCs for 30 minutes or 24 hours after treatment.

Supplementary Figure S16: Cell proliferation and mitotic markers

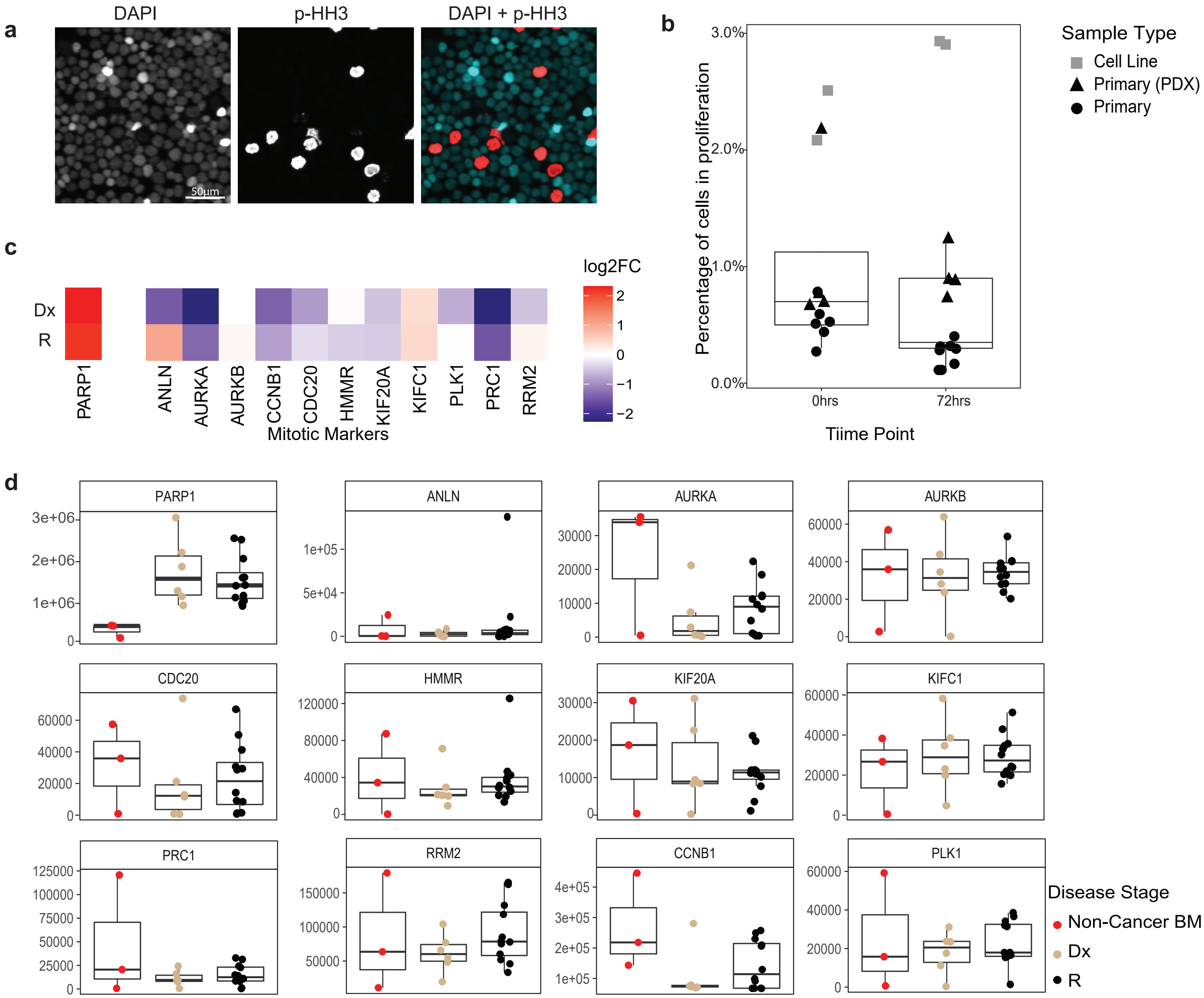

Supplementary Figure S16: Cell proliferation and mitotic markers. a Representative images from the RS411 cell line stained with DAPI and anti-phosphorylated histone H3 to measure proliferating cells at 72 hours at 60x magnification. **b** Summary of the percentage of pHH3 positive cells over the total number of cells for each sample from a single experiment (n= 12 samples at 0hrs and n= 14 samples at 72hrs). Cell lines are represented by grey squares, primary PDX are black triangles, and primary patient samples are black circles. Box represents the interquartile range (IQR), the middle line represents the median and the whiskers extend to 1.5 × IQR. **c** Protein log2 fold-changes for key mitotic kinases and cell cycle regulatory proteins calculated by average protein for Dx (n= 6 patients) or R (n= 11 patients) over the average of the Non-Cancer BM (n= 3 patients). **d** Box plots representing the protein abundance of key mitotic kinases and cell cycle regulatory proteins for Non-cancer BM (red), Dx specimens (tan), and R specimens (black). Box represents the interquartile range (IQR), the middle line represents the median and the whiskers extend to 1.5 × IQR.
