# Supplementary material for: Targetable lesions and proteomes predict therapy sensitivity through disease evolution in pediatric acute lymphoblastic leukemia
Source: Nat Commun. 2023 Nov 21;14:7161. doi: 10.1038/s41467-023-42701-9 (PMC10663560; doi:10.1038/s41467-023-42701-9)
Supplement: Supplementary file 3 — Description of Additional Supplementary Files [file 41467_2023_42701_MOESM3_ESM.pdf]

Description of Additional Supplementary Files

File Name: Supplementary Data 1
Description: BCCH Cohort description

File Name: Supplementary Data 2
Description: BCCH Genomic Data

File Name: Supplementary Data 3
Description: SJH Genomic Data

File Name: Supplementary Data 4
Description: Genomics Progression-Summarized progression analysis of genomic lesions for BCCH and SJH cohorts

File Name: Supplementary Data 5
Description: BCCH-SJH Targets-Summarized druggable events for BCCH and SJH cohorts

File Name: Supplementary Data 6
Description: Cohort-Batch Corrected- Full proteome cohort protein intensities postbatch correction

File Name: Supplementary Data 7
Description: Full Cohort-filtered and imputed- Full proteome cohort protein intensities post-batch correction, filtured and imputed

File Name: Supplementary Data 8
Description: Variable Protein Lists- Supplementary Table S8 variable protein lists used for heatmaps in figure 2 (B&C)

File Name: Supplementary Data 9
Description: B-ALL protein clusters GO

File Name: Supplementary Data 10
Description: Protein Intensities- unfiltered and unimputed- Initial export from Spectronaut of normalized protein intensities prior to filtering and imputation

File Name: Supplementary Data 11
Description: Protein Intensities- filtered and imputed

File Name: Supplementary Data 12
Description: Stats- Paired Summary- Summary of results for statistical analysis of equivalence and difference for paired samples

File Name: Supplementary Data 13
Description: GO equivalent

File Name: Supplementary Data 14

Description: Paired-different log2FC- Summary of log2FC for the proteins that were significantly different in any pair

File Name: Supplementary Data 15
Description: B-cell Analysis- B-cell development transcription factors

File Name: Supplementary Data 16
Description: Cancer Associated Proteins

File Name: Supplementary Data 17
Description: CAP LIMMA

File Name: Supplementary Data 18
Description: RNA-seq BCCH Cohort

File Name: Supplementary Data 19
Description:: RNA-seq TARGET Cohort
