# Supplementary material for: Targetable lesions and proteomes predict therapy sensitivity through disease evolution in pediatric acute lymphoblastic leukemia
Source: Nat Commun. 2023 Nov 21;14:7161. doi: 10.1038/s41467-023-42701-9 (PMC10663560; doi:10.1038/s41467-023-42701-9)
Supplement: Supplementary file 23 — Reporting Summary [file 41467_2023_42701_MOESM23_ESM.pdf]

Corresponding author(s): Dr. Philipp Lange, Dr. Christopher Maxwell

Last updated by author(s): 2023/10/03

Reporting Summary

Nature Portfolio wishes to improve the reproducibility of the work that we publish. This form provides structure for consistency and transparency in reporting. For further information on Nature Portfolio policies, see our Editorial Policies and the Editorial Policy Checklist.

Statistics

For all statistical analyses, confirm that the following items are present in the figure legend, table legend, main text, or Methods section.

n/a	Confirmed	
☐	☒	The exact sample size (*n*) for each experimental group/condition, given as a discrete number and unit of measurement
☐	☒	A statement on whether measurements were taken from distinct samples or whether the same sample was measured repeatedly
☐	☒	The statistical test(s) used AND whether they are one- or two-sided<br>*Only common tests should be described solely by name; describe more complex techniques in the Methods section.*
☒	☐	A description of all covariates tested
☐	☒	A description of any assumptions or corrections, such as tests of normality and adjustment for multiple comparisons
☐	☒	A full description of the statistical parameters including central tendency (e.g. means) or other basic estimates (e.g. regression coefficient) AND variation (e.g. standard deviation) or associated estimates of uncertainty (e.g. confidence intervals)
☐	☒	For null hypothesis testing, the test statistic (e.g. *F*, *t*, *r*) with confidence intervals, effect sizes, degrees of freedom and *P* value noted<br>*Give P values as exact values whenever suitable.*
☒	☐	For Bayesian analysis, information on the choice of priors and Markov chain Monte Carlo settings
☐	☒	For hierarchical and complex designs, identification of the appropriate level for tests and full reporting of outcomes
☐	☒	Estimates of effect sizes (e.g. Cohen's *d*, Pearson's *r*), indicating how they were calculated

Our web collection on statistics for biologists contains articles on many of the points above.

Software and code

Policy information about availability of computer code

Data collection

1) Targeted DNA/RNA fusions were sequenced on the Ion Torrent S5 (ThermoFisher).
2) Samples for RNA-sequencing were prepped and sequenced for the Ion Torrent S5 (Thermofisher)
3) Live cell images were taken using a 40X 0.75 numerical aperture dry objective with the MetaXpress 5.0.2.0 software on the ImageXpress Micro XL epifluorecence microscope (Molecular Devices Inc.)
4)Proteomics data was acquired on an Easy nLC ultra high pressure LC system coupled to Q Exactive HF mass spectrometer (Thermo Scientific) with a µPAC source for liquid chromatography tandem mass spectrometry analysis.
5) Fixed cells were imaged as stacks of 5 optical sections with a spacing of 0.5µm through the cell volume were taken using a 60X 1.2 numerical aperture on an Olympus Fluoview FV10i (Olympus) confocal microscope.

Data analysis	1)DNA/RNA fusions were processed using the Ion Torrent S5 server and analyzed by Ion Reporter version 5.10 with the Oncomine Childhood "Cancer Research - w2.4 - DNA and Fusions - Single Sample" workflow.<br>2) RNA-sequencing data was processed using the Ion Torrent S5 server with the AmpliSeqRNA.html plug in<br>3) Post-acquisition processing of live cell images was performed using MetaXpress offline<br>4) Software and applications used for data analysis include gProfiler (Profiler version<br>570 e104_eg51_p15_3922dba, database updated on 07/05/2021), Cytoscape (version 3.8.2), GraphPad PRISM version 9<br>5) Raw mass spectrometry (MS) files were analyzed with Spectronaut Pulsar X (Biognosys)<br>6) R packages for visualizations and proteomic analysis include "HarmonizR" version, "LIMMA", (version 3.52.2) "pheatmap"(version 1.0.12) "circlize" (version 0.4.15), "dplyr"(version 1.0.9), "ggplot2" (version 3.3.6).<br>7) The immunofluorescence images were analyzed using Fiji-ImageJ.

For manuscripts utilizing custom algorithms or software that are central to the research but not yet described in published literature, software must be made available to editors and reviewers. We strongly encourage code deposition in a community repository (e.g. GitHub). See the Nature Portfolio guidelines for submitting code & software for further information.

Data

Policy information about availability of data

All manuscripts must include a data availability statement. This statement should provide the following information, where applicable:

- Accession codes, unique identifiers, or web links for publicly available datasets
- A description of any restrictions on data availability
- For clinical datasets or third party data, please ensure that the statement adheres to our policy

The raw MS data generated in this study have been deposited to the Proteome Consortium (http://www.proteomexchange.org) via the MassiVE (https://massive.ucsd.edu/) partner repository data set MSV000091012. The raw OCCRA targeted DNA and RNA-fusion sequencing data generated in this study have been deposited to the National Center for Biotechnology information (NCBI) Sequence Research Archive (SRA) (https://www.ncbi.nlm.nih.gov/sra) with the project ID PRJNA985851 and the raw OCCRA RNA-sequencing data generated in this study have been deposited to the NCBI SRA (https://www.ncbi.nlm.nih.gov/sra) with the project ID PRJNA985381. Source data are provided with this paper. The publicly available WES data from 69 pediatric ALL patients from SJH used for the genomics analyses is available in the SJH cloud and can be accessed individually with the patient IDs listed in Supplementary Data 3. The publicly available RNA sequencing data for the 35 pediatric ALL patients from the TARGET cohort was downloaded from the Treehouse Childhood Cancer Initiative at the UC Santa Cruz Genomics Institute, from the data set titled “Tumor cohorts for Vaske et al. publication (October 2019)”. Source data are provided with this paper. The remaining data are available within the Article, Supplementary Information or Source Data file.

Research involving human participants, their data, or biological material

Policy information about studies with human participants or human data. See also policy information about sex, gender (identity/presentation), and sexual orientation and race, ethnicity and racism.

Reporting on sex and gender	Sex was reported for all patients in the study. We investigated bias related to sex but did not notice any trends or bias based on sex. Sex is self-reported by the patient when possible, however with the young age in some cases is reported by the patient's parents or biologically assigned.
Reporting on race, ethnicity, or other socially relevant groupings	Ethnicity was reported for all patients in the study. We investigated bias related to ethnicity but did not notice any trends or bias based on ethnicity. Ethnicity is self-reported by the patient when possible, however with the young age in some cases is reported by the patient's parents.
Population characteristics	Age, sex, clinically reported genetic features, and diagnosis categories are reported for each patient. Clustering analysis performed in the paper demonstrates weak trends based on clinical features. Non-cancer controls were age matched and and evenly representative of sex.
Recruitment	Patients are recruited by trained personnel at the BC Children's Hospital Biobank following predetermined protocols. Patients who undergo bone marrow aspiration for a suspected leukemia or relapse are identified by clinical chart review by trained personnel. The patient's are completely de-indentified by the BioBank during sample collection. There are no known biases affecting patient recruitment, selection of samples, or in analysis.
Ethics oversight	Sample collection and experiments were performed as approved by the University of British Columbia Children & Women’s Research Ethics, and conformed with standards defined in the WMA Department of Helsinki and the Department of Health and Human Services Belmont Report.

Note that full information on the approval of the study protocol must also be provided in the manuscript.

Field-specific reporting

Please select the one below that is the best fit for your research. If you are not sure, read the appropriate sections before making your selection.

☒ Life sciences ☐ Behavioural & social sciences ☐ Ecological, evolutionary & environmental sciences

For a reference copy of the document with all sections, see nature.com/documents/nr-reporting-summary-flat.pdf

Life sciences study design

All studies must disclose on these points even when the disclosure is negative.

Sample size	The sample size was determined by sample availability and is comparable to previously published studies. The caveats associated with sample size are discussed in the manuscript.
Data exclusions	No data was excluded
Replication	1) Protein analysis was acquired in duplicate injections on the mass spectrometer to improve data reproducibility. CVs between replica are presented in the manuscript and all within a reasonable range to demonstrate reproducibility.<br>2) 10 biological replica (6 primary ALL and 4 cell lines) were used for measurement of cytotoxicity in response to targeted inhibitors and acquired by duplicate measurements for each biological sample within 1 experiment. All attempts of replication were successful.<br>3) 30 cells (10 cells each from three images) for each sample was analyzed for measurement of yH2Ax and PARP1 by immunofluorescence, 3 primary ALL biological replica and 2 non-cancer controls. All attempts of replication were successful.<br>4) 23 biological replica (18 primary ALL and 5 non-cancer controls) were used for measurement of cytotoxicity in response to PARP inhibitors and acquired by duplicate measurements for each biological sample with 1 experiment. All attempts of replication were successful.
Randomization	Randomization for allocation into experimental groups is not feasible in this study as the two main groups were cancer vs non cancer, or initial diagnostic samples vs relapsed samples.
Blinding	Blinding was not relevant to this study since experimental groups were assigned based on biological and clinical features.

Reporting for specific materials, systems and methods

We require information from authors about some types of materials, experimental systems and methods used in many studies. Here, indicate whether each material, system or method listed is relevant to your study. If you are not sure if a list item applies to your research, read the appropriate section before selecting a response.

Materials & experimental systems

n/a	Involved in the study	
☐	☒	Antibodies
☐	☒	Eukaryotic cell lines
☒	☐	Palaeontology and archaeology
☒	☐	Animals and other organisms
☒	☐	Clinical data
☒	☐	Dual use research of concern
☒	☐	Plants

Methods

n/a	Involved in the study	
☒	☐	ChIP-seq
☒	☐	Flow cytometry
☒	☐	MRI-based neuroimaging

Antibodies

Antibodies used	yH2Ax (abcam ab2893 at 1:500 dilution)<br>PARP1 (Proteintech 66520-1-Ig at 1:250 dilution)<br>Alexafluor 647 (Invitrogen, A31573, 1:2000)<br>Alexafluor 594 (Invitrogen, A21203, 1:2000)
Validation	yH2Ax (abcam ab2893)- manufacturer states it is batch tested in peptide array, western blot and ICC in mouse fibroblast and rat adrenal gland.<br>PARP1 (Proteintech 66520-1-Ig)- manufacturer states it is validated in multiple cell lines for IHC, IF, WB, IP and FC<br>Alexafluor 647 (Invitrogen, A31573)- manufacturer states it is verified by Relative expression to ensure that the antibody binds to the antigen stated.<br>Alexafluor 594 (Invitrogen, A21203)- manufacturer states it is validated in multiple cell lines for ICC and IF

Eukaryotic cell lines

Policy information about cell lines and Sex and Gender in Research

Cell line source(s)	The following cell lines were used in the study; Nalm6 (M), Jurkat (M) , RS411 (F), BV173 (M), 697 (M). All cell lines were purchased from the American Type Culture Collection (ATCC).
Authentication	All cell lines were purchased directly from ATCC therefore minimal authentication was performed. Authentication of cell lines was done by matching targeted sequencing to published results and ATCC data.
Mycoplasma contamination	Cell lines were not tested for mycoplasma contamination

Commonly misidentified lines<br>(See ICLAC register)	No cell lines were commonly misidentified lines
